# Genome-Wide Analysis Reveals Ancestral Lack of Seventeen Different tRNAs and Clade-Specific Loss of tRNA-CNNs in Archaea

**DOI:** 10.3389/fmicb.2018.01245

**Published:** 2018-06-07

**Authors:** Yue Wu, Ping Wu, Bin Wang, Zhu-Qing Shao

**Affiliations:** ^1^State Key Laboratory of Pharmaceutical Biotechnology, School of Life Sciences, Nanjing University, Nanjing, China; ^2^Institute of Bioinformatics, University of Georgia, Athens, GA, United States

**Keywords:** tRNA copy number, translation efficiency, codon degeneracy, archaeal phylogeny, tRNA modification

## Abstract

Transfer RNA (tRNA) is a category of RNAs that specifically decode messenger RNAs (mRNAs) into proteins by recognizing a set of 61 codons commonly adopted by different life domains. The composition and abundance of tRNAs play critical roles in shaping codon usage and pairing bias, which subsequently modulate mRNA translation efficiency and accuracy. Over the past few decades, effort has been concentrated on evaluating the specificity and redundancy of different tRNA families. However, the mechanism and processes underlying tRNA evolution have only rarely been investigated. In this study, by surveying tRNA genes in 167 completely sequenced genomes, we systematically investigated the composition and evolution of tRNAs in Archaea from a phylogenetic perspective. Our data revealed that archaeal genomes are compact in both tRNA types and copy number. Generally, no more than 44 different types of tRNA are present in archaeal genomes to decode the 61 canonical codons, and most of them have only one gene copy per genome. Among them, tRNA-Met was significantly overrepresented, with an average of three copies per genome. In contrast, the tRNA-UAU and 16 tRNAs with A-starting anticodons (tRNA-ANNs) were rarely detected in all archaeal genomes. The conspicuous absence of these tRNAs across the archaeal phylogeny suggests they might have not been evolved in the common ancestor of Archaea, rather than have lost independently from different clades. Furthermore, widespread absence of tRNA-CNNs in the Methanococcales and Methanobacteriales genomes indicates convergent loss of these tRNAs in the two clades. This clade-specific tRNA loss may be attributing to the reductive evolution of their genomes. Our data suggest that the current tRNA profiles in Archaea are contributed not only by the ancestral tRNA composition, but also by differential maintenance and loss of redundant tRNAs.

## Introduction

Transfer RNA (tRNA) has an important function in protein translation. In this process, tRNAs recognize codons in messenger RNAs (mRNAs) by base pairing between codon and anticodon. They then transfer cognate amino acids onto nascent peptides. The tRNA composition differs among species, which may influence genomic codon usage, translation efficiency, and many other cellular processes (Bermudez-Santana et al., 2010; Michaud et al., 2011; Wang et al., 2011; Shao et al., 2012). Previous studies have provided insights into different factors relevant to the function and evolution of tRNAs, including wobble pairing between tRNA anticodon and synonymous codons, modifications on tRNA anticodons, intron insertion, and *trans*-splicing in tRNA evolution (Randau et al., 2005; Sugahara et al., 2006, 2008; Grosjean et al., 2010; Novoa et al., 2012; Shao et al., 2012).

Wobble pairing and nucleoside modification would extend the recognition spectrum of tRNA, and therefore render functional replacement and tRNA absence in organisms possible. tRNA wobble is a type of relaxed base pairing beyond regular Watson–Crick Pairing between the third position of codon and the first position of tRNA anticodon (Crick, 1966; Gonzalez et al., 1968; Pollak, 1968; Agris, 1991). For instance, G_34_ (the first position of anticodon) in tRNA could pair with U or C at the third position of codon (Grosjean et al., 2010). This kind of pairing makes it possible for tRNA with specific anticodons to detect several variant codons and then replace corresponding tRNAs that specifically recognize these codons. The tRNA wobble could be further influenced by modifications on the first base of anticodon. With necessary modification (cm^5^ U, 5-carboxymethyluridine) by Elp3, tRNA-UNN (U_34_) could also pair with codon NNG in addition to codon NNA, and functionally replace tRNA-CNN (Kurata et al., 2008; Grosjean et al., 2010; Novoa et al., 2012; Selvadurai et al., 2014). Functional redundant tRNAs could actually maintain fewer copies or even be totally lost in some genomes when their cognate codons could be efficiently recognized by other tRNAs via wobble pairing (Grosjean et al., 2010; Novoa et al., 2012). Additionally, some modifications not only affect the wobble ability of tRNAs, but also reduce harmful pairing with irrelevant codons (Näsvall et al., 2007; Huang et al., 2008; Kurata et al., 2008; Grosjean et al., 2010; Novoa et al., 2012; Karlsborn et al., 2014; Selvadurai et al., 2014).

In the past several years, tRNA modification pathway has been thoroughly investigated in both bacteria and eukaryotes, and many modification types have been found (Näsvall et al., 2007; Huang et al., 2008; Kurata et al., 2008; Grosjean et al., 2010). For example, G_34_ that recognizing codons for twofold boxes are frequently replaced by Q_34_ (queuine) and related derivatives (Grosjean et al., 2010), U_34_ could be modified as cmnm^5^U_34_ (5-carboxymethylaminomethyluridine) to stabilize their pairing to guanine (Takai and Yokoyama, 2003; Kurata et al., 2008), or cmo^5^U_34_ (uridine-5-oxyacetic acid) to extend pairing ability (Näsvall et al., 2007). Additionally, for precise detection of AUA codons of isoleucine and avoidance of mistakenly pairing with AUG, IAU (I, inosine) and ΨAΨ (Ψ, pseudouridine) were both used in eukaryotes, while LAU (L, lysidine) was used in bacteria (Grosjean and Björk, 2004; Mandal et al., 2010). In Archaea, a different modification for tRNA-Ile (agmatidine) with the enzyme TiaS has also been reported (Köhrer et al., 2008; Ikeuchi et al., 2010; Mandal et al., 2010). Comparing with bacteria and eukaryotes, for which mechanisms of tRNA modification have been thoroughly investigated, there is still a gap in Archaea with respect to tRNA modification and corresponding enzymes.

In addition to wobble pairing and tRNA modification, degenerative evolution of the genome also drives changes in tRNA composition (Batut et al., 2014). During the process of genome size reduction, some redundant genes tend to be lost, including tRNAs (Oakeson et al., 2014). Functional redundance and driving force from degenerative evolution might both contribute to tRNA evolution of microbe genomes. Additionally, special intron and *trans*-splicing are also exist in tRNAs of archaeal genomes (Marck and Grosjean, 2003). There are increases in the numbers of specific introns in tRNA in Thermoproteales (Sugahara et al., 2008). Separated genes in *Nanoarchaeota equitans* could also combine to produce tRNA (Randau et al., 2005). These two phenomena indicate other possible mechanisms for tRNA evolution (Sugahara et al., 2006; Chan and Lowe, 2016).

Variation in number of introns, fragmentation, permutation, and tRNA arrays, have all been investigated and coevolution between enzymes and tRNA has been suggested (Fujishima and Kanai, 2014; Tran et al., 2015). Most of the diversity in tRNA structure were found in Archaea (Fujishima and Kanai, 2014), suggesting Archaea a useful model for understanding tRNA evolution. In eukaryotes and bacteria, tRNA richness among different genomes has been presented in several previous studies (Duret, 2000; Bermudez-Santana et al., 2010; Grosjean et al., 2010; Michaud et al., 2011). In specific genomes, copy number varies for different tRNA types, and some tRNAs are even totally lost (Gotesson et al., 2002; Xue et al., 2003; Grosjean et al., 2010; Novoa et al., 2012). However, for archaeal tRNAs, past researches on tRNA copy number variation are often less satisfying. They covered few genomes and their presentation and analysis of tRNA composition tend to be sketchy (Novoa et al., 2012). Additionally, these researches are not focused on the tRNAs in the Archaea domain and did not base their analysis on a highly supported phylogenetic tree of archaeal species, reducing confidence on calling of loss or gain events (Grosjean et al., 2010; Novoa et al., 2012).

To present a detailed profile of tRNA composition and evolution in Archaea, a highly supported species tree of Archaea was reconstructed in the present study. The tRNA composition of each genome was analyzed according to this tree. Through these analyses, we found overrepresentation of tRNA-Met in most archaeal genomes, verified extensive absence of tRNA-ANN and tRNA-UAU in Archaea, and investigated some gain events of these types of tRNA. Additionally, we also presented a more extensive analysis of the dense absence of tRNA-CNN in Methanococcales and Methanobacteriales, and discuss the link between genome size and tRNA composition. In addition to extensive tRNA absent, some sparse tRNA gain events were also found among archaeal genomes.

## Materials and Methods

### Data Used in This Study

Here, 167 archaeal genomes and 2 bacterial genomes (*Staphylococcus aureus* NCTC 8325 and *Bacillus subtilis* str 168) were downloaded from the NCBI database^1^. tRNA predictions for the 167 archaeal genomes were downloaded from GtRNAdb (Chan and Lowe, 2016). The list of archaeal genomes used in this study and several genomic features were provided in **Supplementary Table S1**.

### Phylogenetic Analysis

The aforementioned 167 archaeal genomes and 2 bacterial genomes (outgroups) were used for phylogenetic analysis. Proteinortho (Lechner et al., 2011) was used to identify orthologous proteins from these genomes with default parameters. Orthologous groups containing one protein in each genome were selected for phylogenetic analysis. Then each group of orthologous proteins was aligned by ClustalW, Muscle, and Probcons with default parameters, respectively (Edgar, 2004; Do et al., 2005; Larkin et al., 2007). Gblocks was used to select proper regions for phylogenetic analysis and to produce the concatenation with a minimum length of a block of 3, gap position of half and other parameters default. Phylogenetic analysis was done by Phyml (Guindon et al., 2010) using the maximum likelihood (ML) method. The best model for phylogenetic analysis was selected with the Smart Model Selection (SMS) program (Lefort et al., 2017). The aLRT SH-like (Approximate Likelihood Ratio Test Shimodaira-Hasegawa-like) (Anisimova and Gascuel, 2006) value was used to evaluate the reliability of the tree. A flowchart for tree construction was provided in **Supplementary Figure S1**. The alignment from ClustalW was also processed by Gblocks with a minimum length of a block of 10 and subjected to phylogenetic analysis in the same way as described above, to test the robust of the tree with the minimum length of a block of 3.

### Codon Frequency and Optimal Codon Analysis

The frequency of all codons in each genome was calculated separately using an in-house Perl script. Optimal codons were identified using the comparison method as described previously (Wang et al., 2011). For each amino acid, the optimal codon was defined as the most used codon for highly expressed genes. Non-ribosomal proteins with annotations related to transcription and translation processes were selected as high expression genes (**Supplementary Table S6**), whereas all the remaining genes were treated as low expression ones. Ribosomal proteins were not included in optimal codon analysis, as they tend to contain greater numbers of positively charged amino acids (Lott et al., 2013).

### Analysis of tRNA Gains

tRNA sequences gained in archaeal genomes were analyzed by BLAST (Basic Local Alignment Search Tool) (Madden et al., 1996; Camacho et al., 2009) search against the GtRNAdb (Chan and Lowe, 2016) with default parameters. The top five hits with coverage more than 0.5 and with a tRNA annotation were selected, and then aligned by ClustalW (Larkin et al., 2007) in default parameters. R-Coffee with default parameters was used for comparison of the results of tRNA alignment (Notredame et al., 2000; Wilm et al., 2008).

### Analysis of tRNA Modification Enzyme

A total of 29 modification enzymes targeting the first base of tRNA anticodon were selected from Modomics (Ikeuchi et al., 2010; Machnicka et al., 2013; Selvadurai et al., 2014). We first searched these enzymes in annotations of archaeal genomes in NCBI. One of the hit was retrieved as a query sequence to search against all other archaeal genomes with Blastp program to find homologous sequences. The parameters used for blast with an archaeal query include setting *E*-value to 0.001, minimum alignment length to 50% of query length, and minimum percentage of identical matches to 25%. For enzymes that have not been documented in the archaeal annotations in NCBI, sequences from eukaryotes or bacteria were randomly selected for blast search against all archaeal genomes. The parameters used for blast with a query sequence from bacteria or eukaryote include setting *E*-value to 1, minimum alignment length to 40% of query length, and minimum percentage of identical matches to 20%. The reciprocal Blastp was performed to reduce potential false positive and confirm the obtained hits are true orthologs. If one enzyme was detected in a genome, we set the corresponding value for this genome as 1. Otherwise, the value was set to 0.

## Results

### Phylogenetic Analysis of Archaea

**FIGURE 1:**
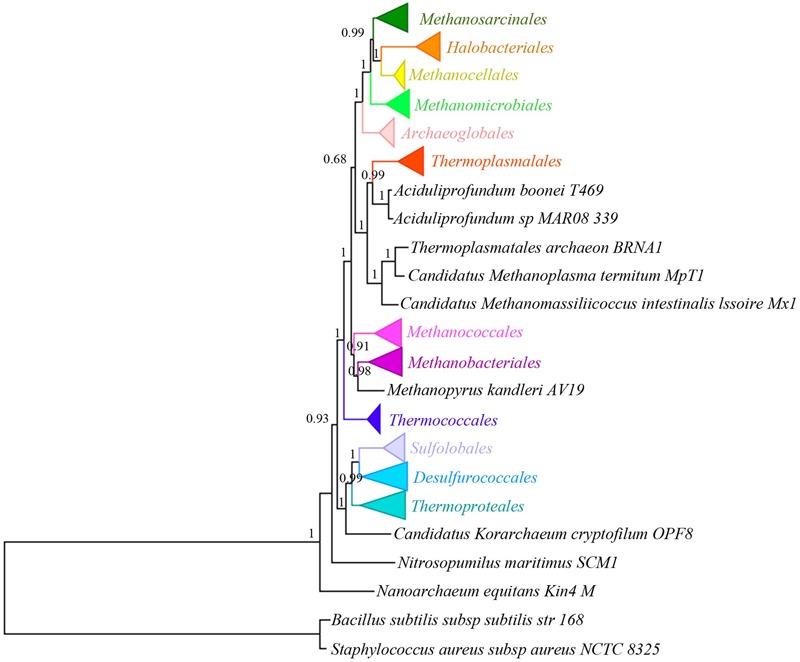
Phylogenetic analysis of 167 archaeal genomes and two bacterial genomes with LG+G+I model. The tree was constructed by ML method using a supermatrix of amino acid sequences (169 × 3509 sites) from ClustalW alignment. Different large clades are in different color. Numbers on the nodes are aLRT SH-like values, which show reliability of corresponding branches. Because some clades have a large number of branches, they are collapsed and the complete tree could be found in **Supplementary Figure S1**.

A total of 20 single copy orthologous proteins were identified from the 167 archaea genomes and 2 bacterial genomes by Proteinortho (Lechner et al., 2011). They consist of 15 ribosomal proteins, including L22, L2, L23, S10, S7, L1, L11, S9, S11, S13, S5, L6, S8, L5, and L14, and several other proteins including methionine tRNA ligase, arginine tRNA ligase, elongation factor 1-alpha, phenylalanine tRNA ligase, and serine/threonine protein kinase. All orthologous groups (each containing 169 protein sequences) were aligned separately. The resulted alignments were then concatenated and processed with Gblock to generate a supermatrix. After selecting proper regions by Gblocks with a minimum length of a block of 3, the three concatenated alignments generated from ClustalW, Muscle, and Probcons are highly consistent in length and gap ratio (**Supplementary Table S2** and **Supplementary Dataset S1**). Therefore, only the one from ClustalW was used for subsequent phylogenetic analysis. The phylogenetic tree was constructed using the ML method with the best model LG+G (Gamma shape parameter: 1.004) +I (invariable sites: 0.057). In the newly constructed tree (**Figure 1** and **Supplementary Figure S1**), five phyla (containing 12 different orders) of Archaea were separated into different lineages, including Crenarchaeota, Euryarchaeota, Thaumarchaeota (only *Nitrosopumilus maritimus* SCM1), Nanoarchaeota (only *Nanoarchaeum equitans* Kin4-M), and Korarchaeota (only *Candidatus Korarchaeum cryptofilum* OPF8). Most branch support value at or above the order level are higher than 0.9, suggesting the topology of the tree is reliable (**Figure 1** and **Supplementary Figure S1**). Furthermore, when we processed the alignment from ClustalW by Gblocks with a minimum length of a block of 10, the resulted tree (**Supplementary Figure S2**) is nearly identical to that with minimum length of a block of 3, with only support value changes at some nodes. This further supported the stable of the tree constructed in this study. Additionally, the tree presented in **Figure 1** (also in **Supplementary Figure S1**) is also quite consistent with those from previous studies (Brochier-Armanet et al., 2011; Zhang et al., 2013). Hence, we will rely on the phylogenetic tree in **Figure 1** for tRNA evolutionary analysis later.

### An Overview of tRNA Distribution in Archaeal Genomes

**FIGURE 2:**
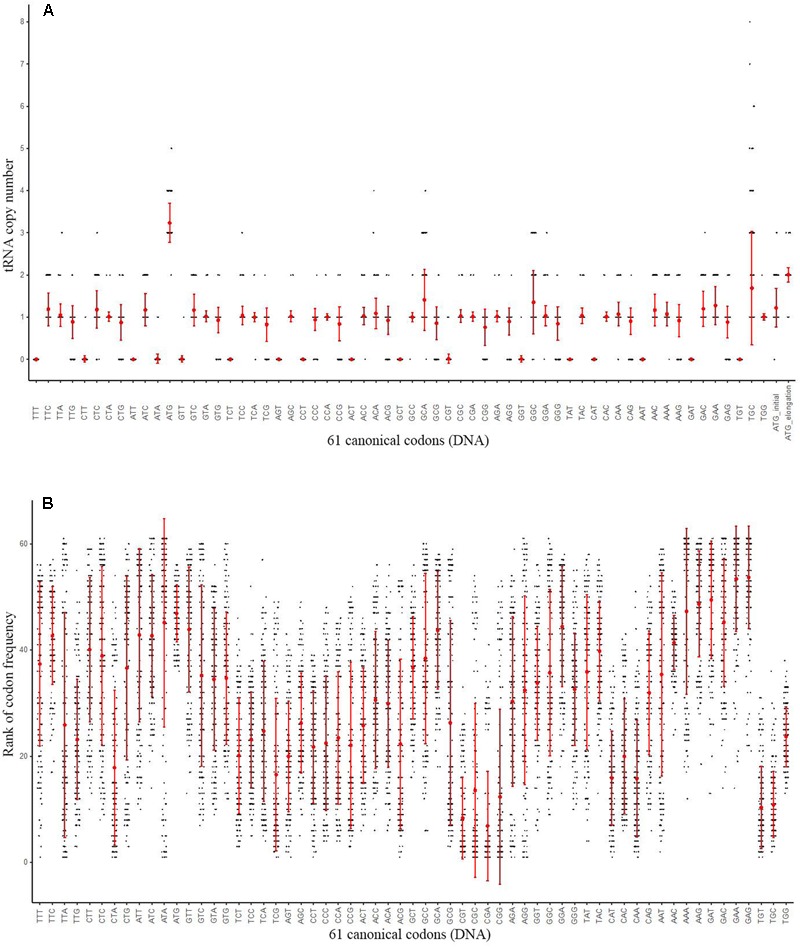
The tRNA copy number and rank of codon frequency for 61 different codons of 167 archaeal genomes. **(A)** tRNA copy number for corresponding codons in Archaea genomes. *X*-axis indicates tRNA types for different codons, while *Y*-axis indicates copy number of tRNA. Copy number for one codon in one species is present as one point. Two types of tRNA pairing Met were also indicated. The error bar of both **(A,B)** indicates standard deviation for each kind of codon or tRNA. **(B)** Rank of codon frequency for 61 codons. *X*-axis indicates codon types, while *Y*-axis indicates frequency rank. More frequent codons are shown with higher rank numbers. The rank of one codon in one species is presented as one point. The order of codon in **(A,B)** are the same.

**FIGURE 3:**
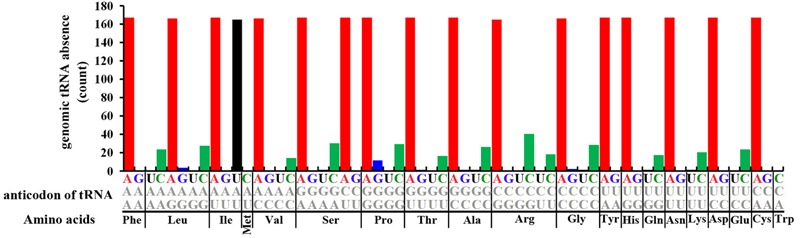
Statistics of absences in different tRNAs in 167 archaeal genomes. The *X*-axis shows different amino acids and anticodons of corresponding tRNAs. The *Y*-axis shows statistics of tRNA loss in 167 Archaea genomes.

An overview of 167 archaeal genomes revealed that Archaea often has low number of tRNA types (**Supplementary Table S3**), with each genome possesses 31 (detected in three genomes) to 45 (*Candidatus Korarchaeum cryptofilum* OPF8) different tRNAs, while for tRNA copy numbers, the range is from 34 (*Methanopyrus kandleri* AV19) to 62 (in four genomes) (**Figure 2A** and **Supplementary Table S3**). While tRNA copy number varies, the diversity of tRNA types was seen only in a small proportion of genomes, with a large proportion of archaeal genomes (104 out of 167) containing 44 different tRNA types (**Figure 2A** and **Supplementary Table S3**). This indicates that there are few types of tRNA in Archaea and the number remains stable for most genomes. The low number of types of tRNA in archaeal genomes suggests that some genetic codons may have no cognate tRNAs to recognize them with Watson-Crick pairing. To determine the distribution of different tRNA types, we further surveyed the copy number of different tRNA types against the 61 genetic codons in each genome (**Figure 3** and **Supplementary Table S3**). As shown in **Figure 3**, tRNAs with anticodon ANNs (tRNA-ANNs) or UAU (tRNA-UAU) are nearly completely absent from all archaeal genomes, while tRNAs with anticodon CNNs (tRNA-CNNs) are also absent in a considerable number of genomes (**Figure 3** and **Supplementary Table S3**). In general, tRNA-CNNs exist in more genomes than tRNA-ANN or tRNA-UAU but fewer genomes than tRNA-UNNs or tRNA-GNNs (**Figure 3** and **Supplementary Table S3**). Additionally, there are also sparse absences for other tRNA types (**Supplementary Table S3**). The absence of many tRNA types may be the major reason of low total tRNA number in archaeal genomes. The copy numbers of tRNAs that are not absent varied only slightly for both different types of tRNA and genomes (**Supplementary Table S3**). Most of them have only one copy per genome on average, although the copy number of specific tRNAs may reach seven in some genomes (**Supplementary Table S3**). Interestingly, the tRNA recognizing codon ATG (tRNA-CAU) is quite abundant among archaeal genomes, with more than three copies on average found in 167 archaeal genomes (**Figure 2A** and **Supplementary Table S3**). To exam whether this uneven distribution of tRNAs in archaeal genomes is correlated with codon usage, we calculated codon frequency of each genome (**Figure 2B**). However, the abundance of tRNA-CAU is not clearly correlated to frequency of ATG codon for 167 Archaea genomes (Pearson correlation, correlation coefficient: -0.04745347, *P*-value = 0.5425) (**Supplementary Figure S3**). Additionally, while there are on average three copies of tRNA-CAU in an archaeal genome, the corresponding codon is not that common among other codons (**Figure 2B** and **Supplementary Table S4**). Ranking the frequency of the codons in each genome rendered it obvious that ATG is never the most common codon in any genome (**Figure 2B**). When we divided tRNA-CAU into those recognizing initial ATG (tRNA-iMet) and elongation ATG (tRNA-eMet), a significantly higher level of tRNA-eMet than other tRNAs (Mean copy number 2.01, *P*-value < 2.2e-16, Mann–Whitney test) was observed. However, there is a quite normal level in tRNA-iMet (**Figure 2A**).

### Widespread Absence of tRNA From Archaeal Genomes

A detailed analysis revealed that 16–31 different types of tRNAs are absent from the 167 genomes, with a maximum of 31 different tRNA types absent from *Methanococcus voltae* A3 and the minimum of 16 different tRNA types absent from *Halalkalicoccus jeotgali* B3 (**Supplementary Table S3**). Most genomes present absence of 17 tRNA types (**Supplementary Table S3**). As described above, the absence of tRNA primarily occurred in tRNA-ANNs, tRNA-UAU and tRNA-CNNs. The absence of tRNA-ANNs or tRNA-UAUs is nearly saturated in archaeal genomes, with a much lower level than other tRNAs (*P*-value < 2.2E-16, Mann–Whitney test) (**Figure 3** and **Supplementary Table S3**). Among the 16 different tRNA-ANNs, 11 were completely absent from all 167 genomes, 3 only presented in one genome, and 2 presented in two genomes (**Supplementary Table S3**). Similarly, the tRNA-UAU gene also only presented in two genomes (**Supplementary Table S3**). Besides tRNA-ANNs and tRNA-UAU, the 16 tRNAs with CNN anticodons are also frequently absent from archaeal genomes, ranging from absence in 14 genomes (absence of tRNA-Val-CAC) to 40 (absence of tRNA-Arg-CCU) (**Supplementary Table S3**). Absence of tRNA-UNNs (except tRNA-UAU) and tRNA-GNNs has also been noted in archaeal genomes, but at very low frequency (**Supplementary Table S3**).

**FIGURE 4:**
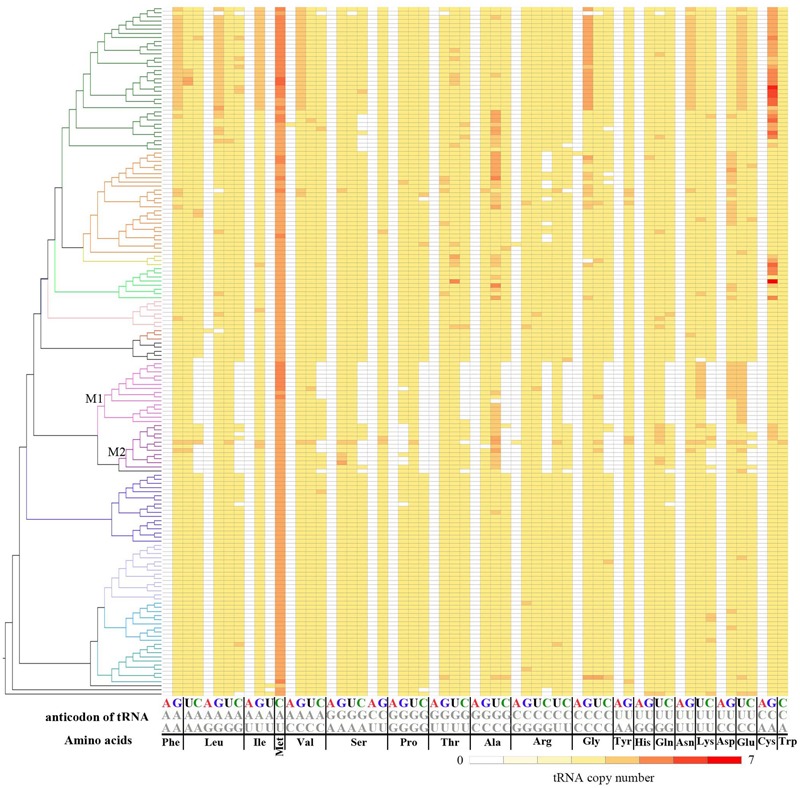
Phylogenetic distribution of different tRNAs in 167 archaeal genomes. The left side of the figure shows a phylogenetic tree of 167 Archaea (**Supplementary Figure S1**), while the right side is copy numbers for tRNA types (column) in studied genomes (row). The column name indicates amino acids and anticodons. Red indicates more tRNA copies and white indicates no corresponding tRNA copies. More details about tRNA number can be found in **Supplementary Table S2**. M1 indicates Methanococcales, and M2 indicates the clade for Methanobacteriales.

After mapping the tRNA number for each codon onto the phylogenetic tree of the 167 genomes, we found that absence of tRNA-CNNs is concentrated in species from Methanococcales and Methanobacteriales. Completely absent of some tRNA-CNNs (tRNA-CAG, tRNA-CAA, tRNA-CGA, tRNA-CGC, tRNA-CCU, tRNA-CUG, tRNA-CUU, and tRNA-CUC) from Methanococcales suggests that gene loss for these tRNAs may have occurred in the common ancestor of Methanococcales. Similarly, completely absent of tRNA-CGG and tRNA-CCG from both Methanococcales and Methanobacteriales suggests that loss of these two tRNAs may have occurred prior to the divergence of the two lineages (**Figure 4**). In contrast, losses of other tRNA-CNNs are often detected in later diverged lineages involving a few species, suggesting loss of these tRNAs may have occurred recently. There is also similar pattern in absence of tRNA-CNNs in *Methanopyrus kandleri* AV19, the only complete genome in Methanopyrales, which is close relative to Methanococcales and Methanobacteriales. Additionally, gene absences across different phylogenetic clades are also more common for tRNA-CNNs, indicating other causes for the more frequent absences (**Supplementary Table S3**).

### Driving Force and Consequence of tRNA Absent From Methanococcales and Methanobacteriales

Our previous study revealed that, tRNA copy number is positively correlated with genome size and genomic GC content in bacteria (Shao et al., 2012). Species experiencing reductive evolution often show reduced genomes and decreased GC content. In Methanococcales and Methanobacteriales, the tRNA copies have greatly reduced compared with other species (the average number of tRNA in Methanococcales and Methanobacteriales is 39.6, while the average number of tRNA in other genomes is 49.4. *P*-value = 2.796e-12, Mann–Whitney test), which is largely due to the widespread absence of tRNA-CNN from the two clades. Analysis of the genomic features of Methanococcales and Methanobacteriales revealed that species in the two clades have far smaller genomes and lower GC contents than species in other clades (the average genome size and GC content in Methanococcales and Methanobacteriales are 1.8 Mb and 0.35, while in other genomes the values are 2.6 Mb and 0.46. *P*-value = 8.619e-05 and 4.2e-11. Mann–Whitney test). However, no clear correlations were found between tRNA copy number (totally and specifically for tRNA-CNN) and those genomic signatures (including genome size and GC content, **Supplementary Figure S3**) among all archaeal genomes. To test whether tRNA variation has shaped the codon usage in Methanococcales and Methanobacteriales, we calculated the frequency of all genetic codons in each genome. Methanococcales and Methanobacteriales had significantly lower proportion in NNG codons for all twofold, fourfold and sixfold amino acids (**Supplementary Tables S4, S5**). Furthermore, the optimal codons were identified form all Archaeal genomes (**Supplementary Tables S6, S7**), which revealed that the frequency of NNGs as optimal codons was also significantly lower for Methanococcales and Methanobacteriales species (*P*-value = 3.089e-07, Mann–Whitney test, **Supplementary Table S8**).

To determine whether the tRNA composition variation among Archaeal genomes is related to modification on anticodon region, the homologs of 29 known tRNA modification enzymes within archaeal genomes were analyzed (**Supplementary Dataset S2**). There is a diverse pattern of enzyme existences among archaeal genomes. While some proteins, such as TilS and Elp3, are present in most genomes, other enzymes tend to be absent from some genomes (**Supplementary Table S9**). However, there is no evidence for specific absence of modification enzyme from Methanococcales and Methanobacteriales to explain the absence of tRNA-CNN.

### Detection of tRNA Gains in Archaeal Genomes

In addition to the absence of several tRNAs from many genomes, we also detected gain of tRNAs for different codons from several genomes. Because tRNA-ANNs and tRNA-UAUs are nearly completely absent from all 167 genomes, the identification of tRNA-Leu-AAG in *Ferroplasma acidarmanus* fer1 genome, tRNA-Val-AAC in *Methanohalobium evestigatum* Z-7303 genome, tRNA-Gly-ACC in *Natrinema pellirubrum* DSM 15624 genome, tRNA-Ile-UAU in *Candidatus Korarchaeum cryptofilum* OPF8 and *Nanoarchaeum equitans* Kin4-M genomes, tRNA-Arg-ACG in *Halalkalicoccus jeotgali* B3 and *Methanobrevibacter ruminantium* M1 genomes indicate species-specific gain of these tRNAs (**Figure 4** and **Supplementary Table S3**).

**FIGURE 5:**
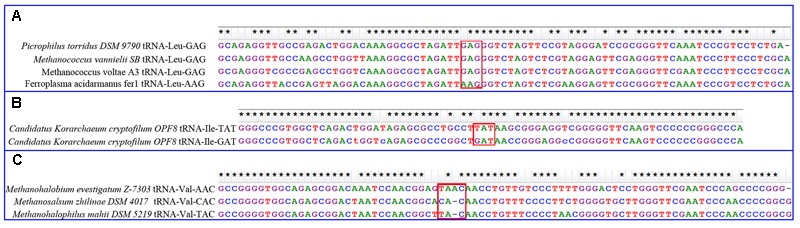
Alignment of gained tRNAs and the best BLAST hits. **(A)** Alignment of leucine tRNA-AAG in *Ferroplasma acidarmanus* fer1 genome and three other BLAST hits. They are all without intron. **(B)** Alignment of isoleucine tRNA-UAU in *Candidatus Korarchaeum cryptofilum* OPF8 genome and a BLAST hits. The former has an intron, which is removed before alignment. **(C)** Alignment of valine tRNA-AAC and two BLAST hits. They all have introns, which are removed before alignment. Stars at the top indicate sequences identical at corresponding sites. Red boxes are predicted sites for anticodons. The alignments are produced by ClustalW with default parameters.

To determine possible mechanisms underlying these tRNA gains, BLAST search was performed against the GtRNAdb database. Using tRNA-Leu-AAG from *Ferroplasma acidarmanus* fer1 as a query, three high-confidence hits from *Picrophilus torridus* DSM 979, *Methanococcus vannielii* S and *Methanococcus voltae* A3 were obtained, all of which were leucine tRNAs with the anticodon GAG (**Figure 5**). Additionally, tRNA-GAG was absent from *Ferroplasma acidarmanus* fer1 and present in all closely related genomes in Thermoplasmatales. In this way, tRNA-Leu-AAG in *Ferroplasma acidarmanus* fer1 genome might be caused by nucleotide substitution from G to A in the first position of anticodon of the pre-existing tRNA-Leu-GAG. Similarly, tRNA-Val-AAC in *Methanohalobium evestigatum* Z-7303 genome was found to be attributable to one base insertion at the anticodon of tRNA-Val-TAC by ClustalW (**Figure 5**) and one base substitution by R-Coffee (**Supplementary Dataset S3**). The best hit of tRNA-Ile-UAU in *Candidatus Korarchaeum cryptofilum* OPF8 is another isoleucine tRNA with anticodon GAU in the same genome (**Figure 5**). Besides the difference in anticodon sequences, there is one intron in tRNA-UAU but no intron in tRNA-GAU. After removal of the intron, these sequences are quite consistent. The three gains of new tRNAs are all attributable to mutations in pre-existing homologous tRNAs with other anticodons. For the other four gains of tRNAs, blast search obtained no high confident hits, so their origins could not be determined.

To explore whether gain of these tRNAs have affected the usage frequency of their cognate codons, we compared usage frequencies of these codons between these genomes and their most closely relatives on the tree. Interestingly, we did not see enhanced frequency for these codons (**Supplementary Table S10**) in the genome of *Ferroplasma acidarmanus* fer1, *Methanohalobium evestigatum* Z-7303, *Natrinema pellirubrum* DSM 15624 genome, *Halalkalicoccus jeotgali* B3 and *Methanobrevibacter ruminantium*, when comparing with the genomes of their closely related species on the tree. This suggested that gain of tRNA has no detected effect on the usage of their cognate codons. The possible reason is that the gene gain events may have occurred very recently, and it needs a long time to alter the genomic codon usage. For *Candidatus Korarchaeum cryptofilum* OPF8 and *Nanoarchaeum equitans* Kin4-M, they each forms a single lineage on the tree, and therefore we could not evaluate their codon usage variation due to lack of information from closely related species.

## Discussion

### Comparison of Archaeal Phylogenetic Trees

Phylogenetic tree in this study was constructed with the best model (LG+G+I) and the topology is well supported. The topology of the tree is also quite similar to those of previous works (Brochier-Armanet et al., 2011; Zhang et al., 2013), especially in the relationship and contents of orders. One of the differences between our and previous studies is the relationship among Methanomicrobiales, Methanosarcinales, Methanocellales, and Halobacteriales, which has not been found to be stable in previous studies (Brochier-Armanet et al., 2011; Zhang et al., 2013). In the newly constructed tree (**Figure 1**), Halobacteriales and Methanocellales form a clade (aLRT SH-like value = 1). The clade then forms a larger clade with Methanosarcinales (aLRT SH-like value = 0.99), and a even larger clade with Methanomicrobiales (aLRT SH-like value = 1). However, the phylogenetic relationship for the four orders is different between the present study and the two previous studies. For example, Halobacteriales is closely related to Methanomicrobiales in the result of Brochier-Armanet et al. (2011), whereas Methanomicrobiales is closely related to Methanosarcinales in the result of Zhang et al. (2013). It seems that reconstruction of the relationship among the four groups is unstable and difficult, even though there are already many genomes sequenced (71 used in this study). This difficulty might be attributable to the close phylogenetic relationship of these species. Because most parts of the phylogeny are identical to those from previous studies, presenting and analyzing tRNA distribution according to this new tree is feasible.

### tRNA Distributions in Archaea

An extensive analysis of tRNA copy number distribution of archaeal genomes is an important supplement to the facts found in tRNA structure evolution. They both contribute to understanding of translation process (Fujishima and Kanai, 2014). Our study revealed a highly stable distribution of tRNA copies, though there is variance sparsely or restricted to some clades, such as Methanosarcinales, Halobacteriales, Methanococcales, and Methanobacteriales. However, the copy number of tRNA-CAU tends to be higher among archaeal genomes, with an average three copies and it seems not correlated to codon frequency in general. For the two different types of tRNA-CAU, the initiation tRNA has a normal copy number, while the elongation tRNA has a significantly higher number. Hence, summing of two functions and increase of copy number in one type could both explain more copies of tRNA-CAU (Kolitz and Lorsch, 2010). One possible hypothesis for the higher number of tRNA-eMet is that the cytosine at the anticodon could be modified to agmatidine. This modified tRNA could function as tRNA-Ile and efficiently recognize codon AUA, which could explain both high level of tRNA-CAU and absence of tRNA-UAU in Archaea (Köhrer et al., 2008; Mandal et al., 2010, 2014). Some other hypotheses include more copies to ensure translation efficiency and relieve possible competence between two types of tRNA-CAU. Though translation initiation is a rate-limiting step in translation, improving translation speed could not further explain the high number of tRNA-CAU (Reeve et al., 2014). Considering we have detected TiaS enzyme from a large proportion of archaeal genomes, we speculate that modification of tRNA-CAU to recognize codon AUA is the major drive force for its high copy number.

### tRNA Absences From Archaea by Different Mechanisms

From the results, it could be seen that tRNA-ANNs and tRNA-UAUs are extensively absent in Archaea, while tRNA-CNNs are mainly absent from Methanococcales and Methanobacteriales, which is consistent with previous research on fewer species from these two clades (Grosjean et al., 2010; Novoa et al., 2012). The widespread presence of tRNA-CNNs across the archaeal phylogeny outside Methanococcales and Methanobacteriales clades suggests that these absences could be attributing to tRNA loss. tRNA loss could be explained by limits and driving forces. The former means whether tRNA could be replaced functionally and so be lost, including tRNA wobbling, and the existence and efficiency of modification enzymes (Grosjean et al., 2010; Novoa et al., 2012), while the latter means that degenerative evolution may drive tRNA loss. In Archaea, after modification by Elp3 and efficiency increase for pairing with G at the third position of the codon, tRNA-UNN could functionally replace tRNA-CNN (Karlsborn et al., 2014; Selvadurai et al., 2014). Existence of Elp3 in many archaeal genomes also indicates general possibility of this replacement (**Supplementary Table S9**). Hence, tRNA-CNNs could be functionally replaced and may be lost in Archaea. Degenerative evolution could reduce the size of the genome and cause gene loss. If some types of tRNA could be functionally replaced, this driving force could also cause them to be lost. A correlation between genome size and tRNA copy number has been observed in both bacteria and eukaryotes (Bermudez-Santana et al., 2010; Michaud et al., 2011). There is a possible link between reduced genome size and absence of tRNA-CNNs from Methanococcales and Methanobacteriales. In these two groups, both genome size and tRNA copies were reduced. However, more detailed analysis and experimental evidence are needed to clearly establish the specific driving forces in corresponding environments.

Absence patterns differ between tRNA-ANNs and tRNA-CNNs. In Archaea, while tRNA-CNNs tend to be absent from limited clades and primarily caused by clade-specific tRNA loss in Methanococcales and Methanobacteriales, tRNA-ANNs are nearly completely absent from archaeal genomes. Because the functional replacement of tRNA-ANNs by tRNA-GNNs does not depend on any enzymes (Grosjean et al., 2010; Novoa et al., 2012; Karlsborn et al., 2014; Selvadurai et al., 2014), two alternative hypotheses would explain the absence of tRNA-ANNs in archaeal genomes. One is that the Last Universal Common Ancestor (LUCA) had evolved a full set of 61 genetic codons, but the tRNA-ANNs are lost in the common ancestor of Archaea. Another hypothesis is that the LUCA had only the minimum set of tRNAs (44 or 45 different types) to decode all codons before the appearance of the three life domains (van der Gulik and Hoff, 2016). The types of tRNA for most archaeal genomes across different phylogenetic clades are coincident with this number. Therefore, the absent of tRNA-ANNs in archaeal genomes suggested these types of tRNAs may have not been evolved in Archaea at all. Because the functional replacement of tRNA-ANNs does not rely on tRNA modification, both of the above hypotheses are possible. However, the first hypothesis requires the ancestral Archaea first increases its tRNA set from 44 or 45 (in the LUCA) to 61 and then decreases to 44 again. This requires multiple independent tRNA gain and loss events to delete all 16 different types of tRNA-ANNs. Therefore, the later hypothesis seems more parsimonious and probably to occur. Taken together, the widespread absence of tRNA-ANNs and tRNA-UAU in Archaea and clade-specific absence of tRNA-CNNs in Methanococcales and Methanobacteriales have experienced totally different stories in evolution.

### tRNA Gains in Archaea

Several gains of tRNA-ANN and tRNA-UAU are also analyzed in this study. Among the seven tRNA gain events, three are traceable, which results from gene duplication, intron insertion, nucleotide substitution, and nucleotide insertion into the anticodon region. Previous studies have shown that mutations in the anticodon are sufficient to alter the specificity of a tRNA in both bacteria and eukaryotes (Saks et al., 1998; Rogers and Griffiths-Jones, 2014). This study provides evidence that these types of alterations in tRNA specificity occurred in Archaea naturally. In addition to high-confidence results, most BLAST results only cover about half of the tRNA sequence, indicating the role of gene combination, such as *trans*-splicing, in tRNA evolution (Randau et al., 2005). Gene combination could also explain scarceness of tRNA homolog. Additionally, because some BLAST results are tRNA genes from other species or even other life domains, HGT might also be important to tRNA evolution (Tuller, 2011; Knie et al., 2015). Detection and clarification of tRNA gain events beyond tRNA-ANNs and tRNA-UAU require more genome sequences and more comprehensive evolution analysis. Detection of tRNA gains in seven genomes suggests that the evolution of tRNA in Archaea is rather dynamic. However, the effect of tRNA gain may have been underestimated due to the uncertainty surrounding tRNA gain events beyond tRNA-ANNs and tRNA-UAU.

## Conclusion

In this study, we traced the evolutionary history of tRNAs in Archaea under the phylogenetic background and presented several novel findings. Firstly, we detected prevalent overrepresentation of tRNA-eMet across different archaeal genomes. Secondly, our results support that the tRNA-ANNs might have not been evolved in the common ancestor of Archaea. In comparing, widespread absence of tRNA-CNNs is attributed to the convergent and ongoing loss of these tRNAs in the two clades. Third, we detected tRNA-UAU for Ile from two Archaeal species. This type of tRNA has been suggested to absent in Archaea. Additionally, taking the advantage of the large number of genomes used in this study and the well-supported phylogenetic tree, we identified several tRNA gain events in archaeal genomes. These findings provide novel insights into the evolution of tRNAs in Archaea.

## Author Contributions

Z-QS, BW, and YW designed the project. YW, PW, and Z-QS carried out the analysis. Z-QS, BW, and YW did the discussion. YW draft the manuscript. Z-QS modified the manuscript.

## Conflict of Interest Statement

The authors declare that the research was conducted in the absence of any commercial or financial relationships that could be construed as a potential conflict of interest.

## Footnotes

1 http://www.ncbi.nlm.nih.gov

## Supplementary Material

FIGURE S1 ML tree of 167 archaeal genomes and two bacterial genomes with LG+G+I model. Different orders or families are indicated with different colors. Numbers on nodes are the aLRT SH-like value. The sequences are aligned by ClustalW that processed by Gblocks with a minimum length of a block of 3. A workflow diagram was also listed in the figure.

FIGURE S2 ML tree of 167 archaeal genomes and two bacterial genomes with LG+G+I model. Different orders or families are indicated with different colors. Numbers on nodes are the aLRT SH-like value. The sequences are aligned by ClustalW that processed by Gblocks with a minimum length of a block of 10.

FIGURE S3 Correlation between genomic character and tRNA copy number. Correlation among GC content, Genome size, tRNA type number, tRNA-CNNs type number, proportion of NNG codon, and number of optimal codon as NNG (shown as NNG in the figure). Correlation and *P*-value are indicated in the figure. Correlation is in front of the slash, while *P*-value is behind it.

TABLE S1 Genome size and GC content of 167 analyzed genomes.

TABLE S2 Identity and gap ratio of alignments for ClustalW, Muscle and Probcons.

TABLE S3 tRNA composition of all archaeal genomes. Different colors present different orders or families as indicated in **Supplementary Figure S1**. The columns on the right side show predicted copy number of tRNA gene (with repeat), predicted number of tRNA gene type (that is numbers of anticodons), predicted number of absent tRNA, predicted copy number of tRNA with anticodon CNNs, ANNs, UNNs, and GNNs of corresponding genomes.

TABLE S4 Codon proportion in all genes of 167 archaeal genomes. Different colors present different orders or families as indicated in **Supplementary Figure S1**. The frequency was the ratio between codon frequency and sum of codon frequency for the same amino acid. The rows indicate different genomes, while the columns indicate different codon types.

TABLE S5 NNGs codon usage for different fold boxes in 167 archaeal genomes. The rows indicate codon folds for amino acid, while the columns indicate different group, including Methanococcales and Methanobacteriales, and other groups. The *P*-value was drawn by Mann–Whitney test.

TABLE S6 High expression gene list used for optimal codon prediction.

TABLE S7 Optimal codon for different amino acid in archaeal genomes. The optimal codon was defined as the most used codon for corresponding amino acid in high expression genes. The rows indicate different genomes, while the columns indicate different amino acid. Different colors indicate codon with different third position nucleotide.

TABLE S8 Difference of NNGs, NNAs, NNCs, and NNUs as optimal codons in Methanococcales and Methanobacteriales, and other genomes. The rows indicate the two different groups, while the columns indicate different optimal codon types. This table is based on information of **Supplementary Table S5**.

TABLE S9 Distribution of 29 modification enzymes in Archaea. A total of 29 enzymes related to the modification on the first position of anticodon of tRNA were analyzed in Archaea. 0 indicates no evidence for existence of corresponding enzyme or similar genes in the genome, while 1 indicates the evidence of existence.

TABLE S10 The frequency of codons cognate to gained tRNAs in five species and their closely relatives.

DATASET S1 Alignments for phylogenetic analysis by ClustalW, Muscle, and Probcons. This zip file includes concatenated alignment of orthologous proteins used in phylogenetic analysis. Single copy proteins were selected from all orthologous groups and were aligned separately by ClustalW, Muscle, and Probcons. Gblocks was used to select proper region for phylogenetic analysis and to produce the concatenation with minimum length of a block 3, allowed gap position half, and other parameters default.

DATASET S2 Sequences of 29 tRNA modification enzymes used as query sequence in this study.

DATASET S3 Alignment result for gained tRNA by R-Coffee. This zip file includes alignment of three gained tRNA and corresponding best BLAST hit. All introns were deleted before align. The alignments are produced by R-Coffee with default parameters.
