# Supplementary figures and images for: Genome-Wide Analysis Reveals Ancestral Lack of Seventeen Different tRNAs and Clade-Specific Loss of tRNA-CNNs in Archaea
Source: Front Microbiol. 2018 Jun 7;9:1245. doi: 10.3389/fmicb.2018.01245 (PMC6000648; doi:10.3389/fmicb.2018.01245)

# A flowchart for tree construction

## Procedure

## Software

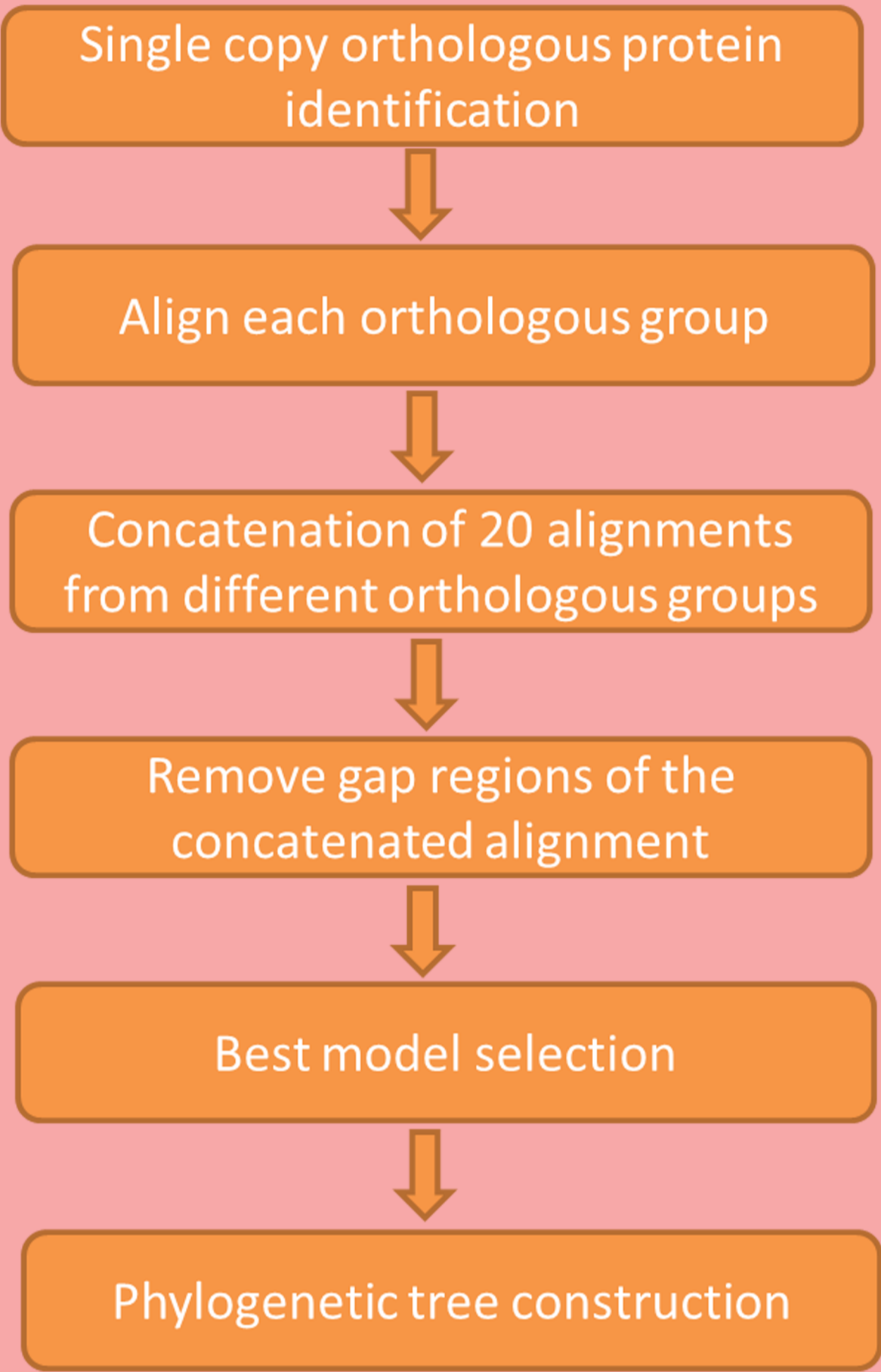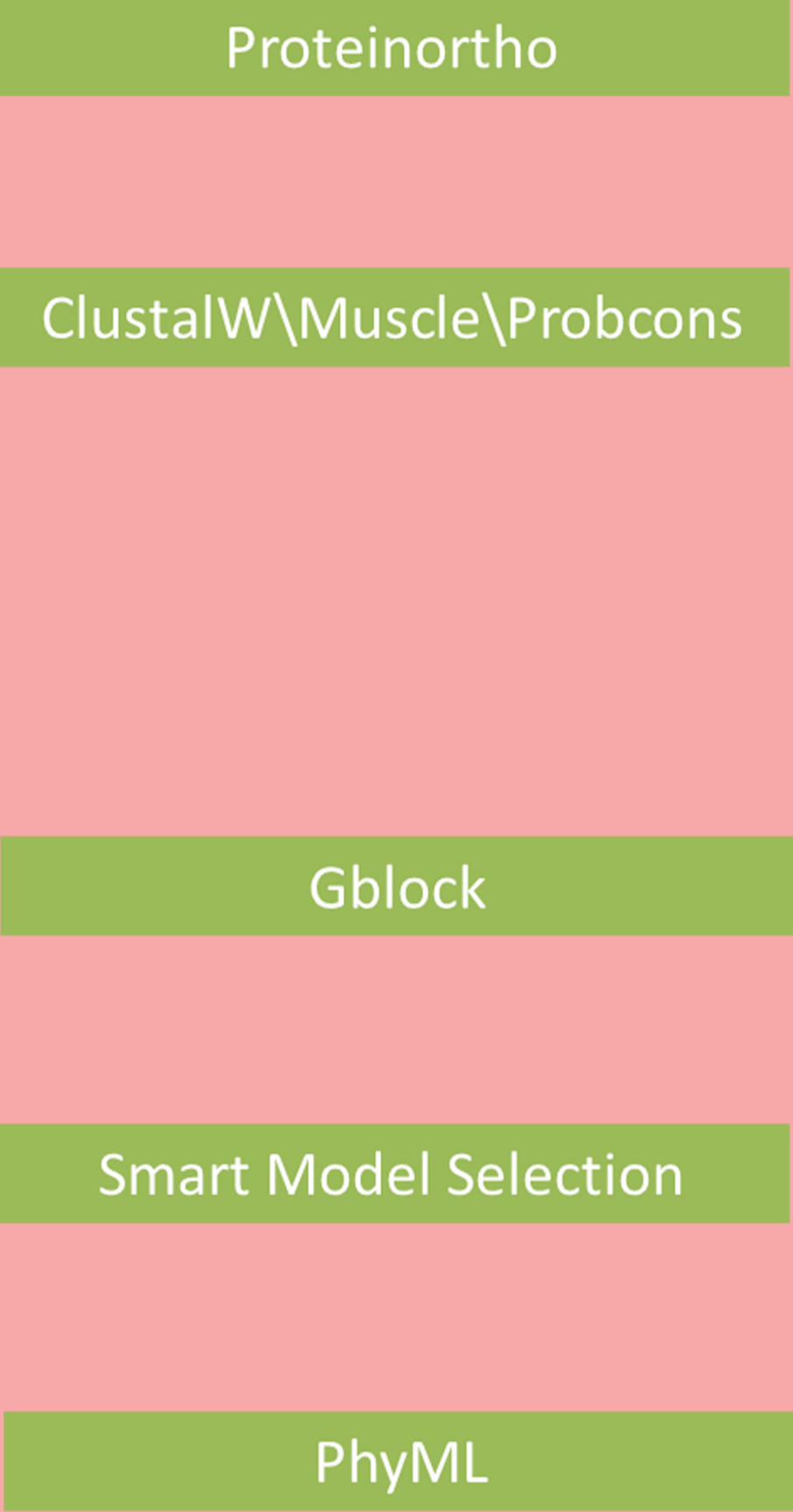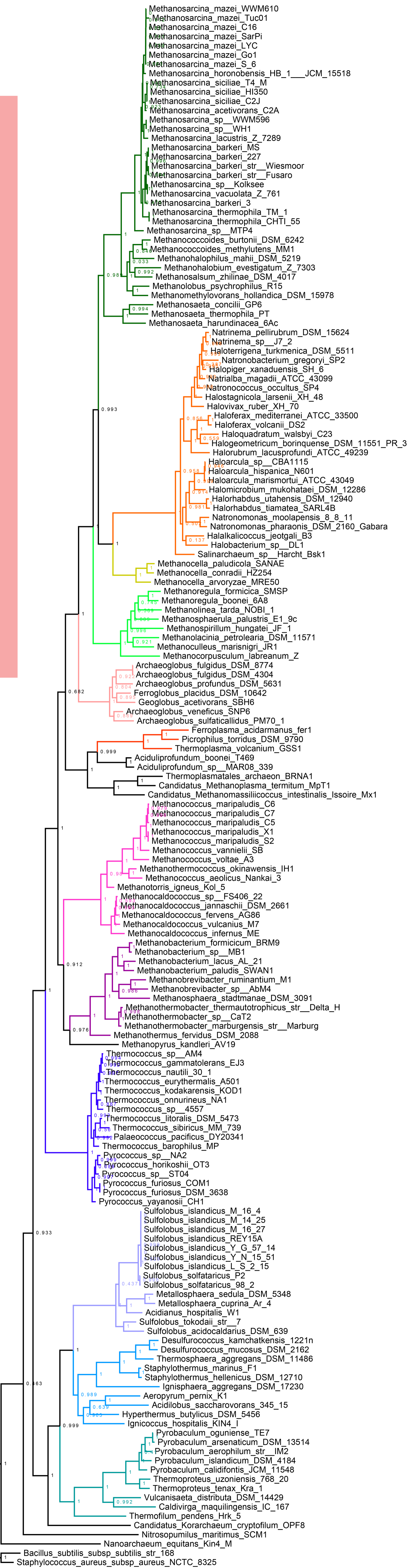

Supplement: FIGURE S1 — ML tree of 167 archaeal genomes and two bacterial genomes with LG+G+I model. Different orders or families are indicated with different colors. Numbers on nodes are the aLRT SH-like value. The sequences are aligned by ClustalW that processed by Gblocks with a minimum length of a block of 3. A workflow diagram was also listed in the figure. [file Image_1.PDF]

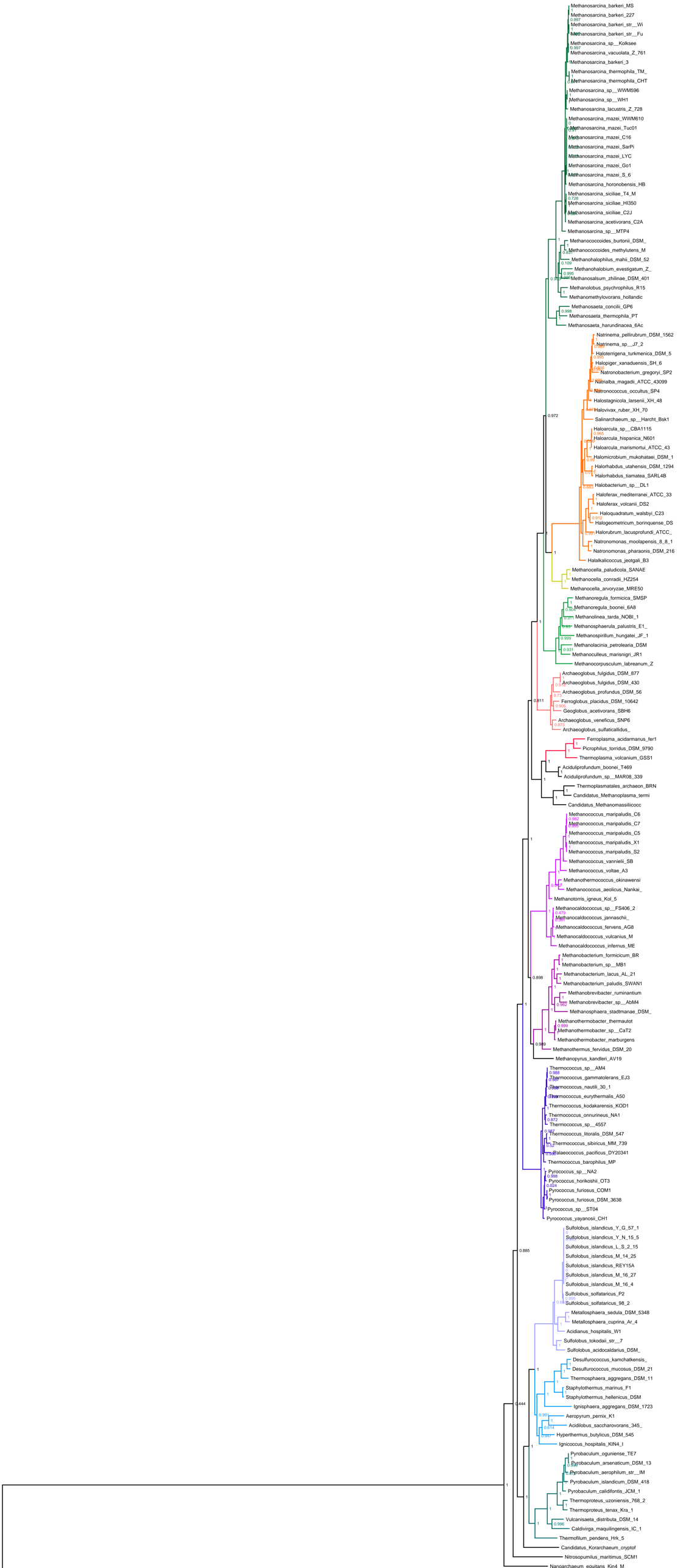

Supplement: FIGURE S2 — ML tree of 167 archaeal genomes and two bacterial genomes with LG+G+I model. Different orders or families are indicated with different colors. Numbers on nodes are the aLRT SH-like value. The sequences are aligned by ClustalW that processed by Gblocks with a minimum length of a block of 10. [file Image_2.PDF]

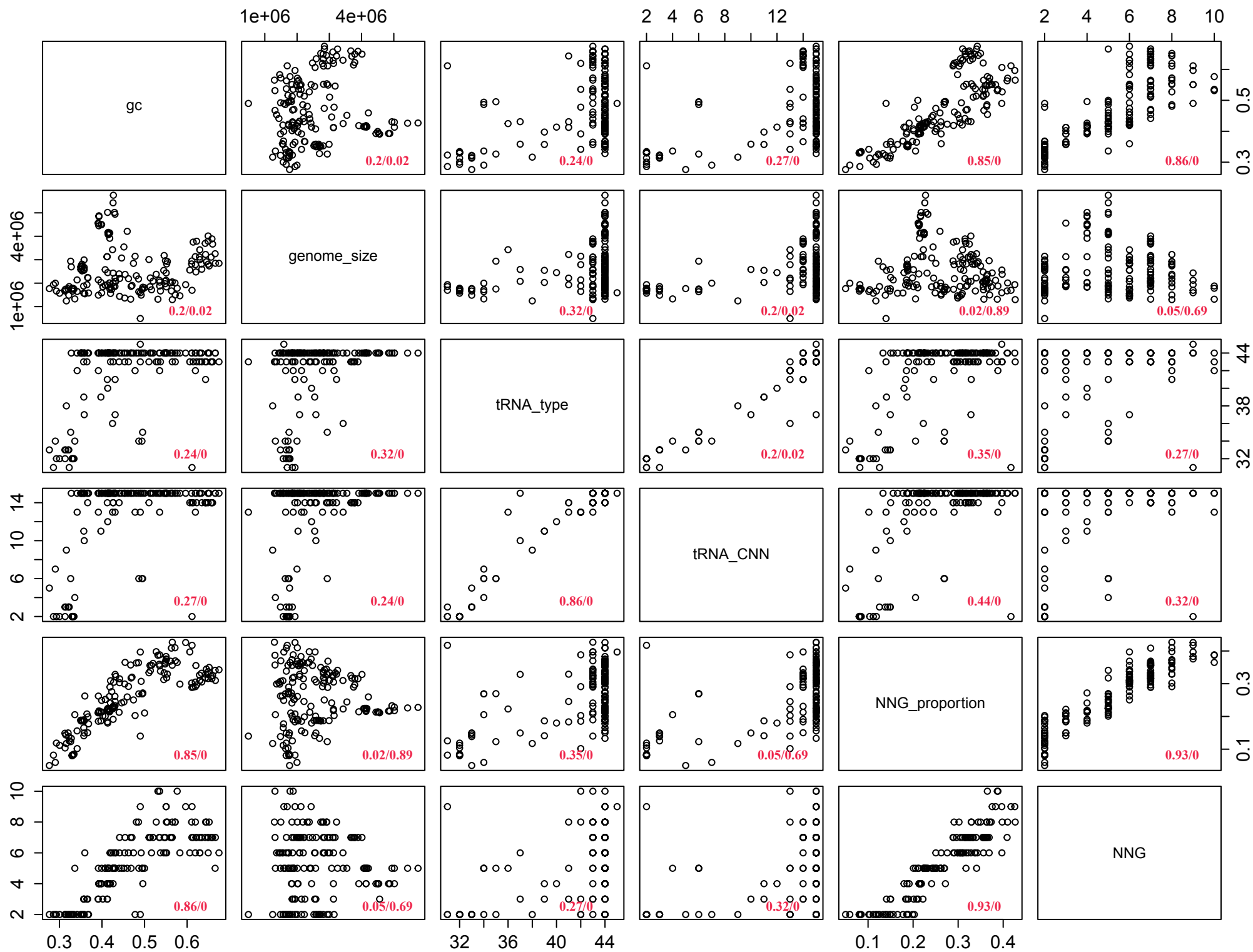

Supplement: FIGURE S3 — Correlation between genomic character and tRNA copy number. Correlation among GC content, Genome size, tRNA type number, tRNA-CNNs type number, proportion of NNG codon, and number of optimal codon as NNG (shown as NNG in the figure). Correlation and P-value are indicated in the figure. Correlation is in front of the slash, while P-value is behind it. [file Image_3.PDF]
